# EVERYDAY FUNCTION PROFILES IN PRODROMAL STAGES OF MOTORIC COGNITIVE RISK SYNDROME: A PROSPECTIVE COHORT STUDY

**DOI:** 10.1093/geroni/igac059.2962

**Published:** 2022-12-20

**Authors:** Nigel Kravatz, Dristi Adhikari, Emmeline Ayers, Joe Verghese

**Affiliations:** Albert Einstein College of Medicine, Bronx, New York, United States; Albert Einstein College of Medicine, Bronx, New York, United States; Albert Einstein College of Medicine, Bronx, New York, United States; Albert Einstein College of Medicine, Bronx, New York, United States

## Abstract

Assessment of everyday function is crucial to diagnosing pre-dementia and dementia syndromes in older individuals. Prodromal stages of mild cognitive impairment (MCI) are associated with mild limitations in complex activities of daily living. It is unknown whether such limitations exist in the prodromal period before criteria is met for motoric cognitive risk syndrome (MCR), a pre-dementia syndrome defined by subjective cognitive complaints and slow gait speed. We compared complex everyday functional profiles at baseline in 46 community-dwelling older individuals (aged 65+) with normal cognitive performance at baseline who went on to develop incident MCR (‘pre-MCR’) with 265 older individuals who remained cognitively normal over follow-up. Patients diagnosed with MCI and global cognitive function scores more than 1.5 standard deviations below the mean at baseline were excluded. The mean number of limitations on complex everyday function at baseline was 3.52±3.1 in the pre-MCR cases and 1.78±2.2 in the 265 normal controls (OR 1.28; 95% CI 1.14–1.43). After adjusting for demographic factors, comorbidities, and follow-up time, the overall number of limitations remained significantly higher in the pre-MCR participants compared to the normal participants (OR 1.18; 95% CI 1.02–1.37). Of the limitations, difficulty completing hobbies and handling finances were associated with pre-MCR after adjustments. Limitations on complex everyday functions in individuals occur before they meet criteria for incident MCR on longitudinal follow-up. Such limitations over time will help clinicians identify individuals at-risk for dementia early in clinical practice.

